# Management of Adult Patients with Primary Immune Thrombocytopenia (ITP) in Clinical Practice: A Consensus Approach of the Spanish ITP Expert Group

**DOI:** 10.1155/2019/4621416

**Published:** 2019-08-22

**Authors:** M. Eva Mingot-Castellano, M. Teresa Álvarez Román, Luis Fernando Fernández Fuertes, Tomás José González-López, José María Guinea de Castro, Isidro Jarque, M. Fernanda López-Fernández, Maria Luisa Lozano, Blanca Sánchez González, David Valcárcel Ferreiras, José Ramón González Porras

**Affiliations:** ^1^Service of Hematology, Hospital Virgen del Rocio, Sevilla, Spain; ^2^Service of Hematology, Hospital Universitario La Paz, Madrid, Spain; ^3^Service of Hematology, Complejo Hospitalario Universitario Insular Materno-Infantil, Las Palmas de Gran Canaria, Spain; ^4^Service of Hematology, Hospital Universitario de Burgos, Burgos, Spain; ^5^Service of Hematology and Hemotherapy, Universitario de Araba, Vitoria-Gasteiz, Álava, Spain; ^6^Service of Hematology and Hemotherapy, Hospital Universitario y Politécnico La Fe, Valencia, Spain; ^7^Service of Hematology and Hemotherapy, Instituto de Investigación Biomédica de A Coruña (INIBIC), Complejo Hospitalario Universitario A Coruña, A Coruña, Spain; ^8^Department of Hematology and Medical Oncology, Hospital General Universitario Morales Meseguer, IMIB-Arrixaca, CIBERER, Murcia, Spain; ^9^Service of Clinical Hematology, Hospital del Mar-Parc de Salut Mar, Barcelona, Spain; ^10^Hematopoietic Stem Cell Transplantation Unit, Hospital Universitario Vall d'Hebron, Barcelona, Spain; ^11^Service of Hematology, Hospital Universitario de Salamanca-IBSAL, Salamanca, Spain

## Abstract

**Background and Objective:**

Diagnosis and management of primary immune thrombocytopenia (ITP) have changed dramatically in the last decade. The aim of the study was to obtain information about the opinion of the Spanish ITP Group (GEPTI) members regarding the best clinical practices for diagnosis and management of adult patients with ITP.

**Materials and Methods:**

A two-round Delphi method was carried out by sending to 129 experts a 90-item questionnaire developed by 11 specialists, with a 4-point Likert scale (“never,” “sometimes,” “frequently,” and “always”) for the assessment of responses.

**Results:**

Forty out of the 129 experts participated in the survey (participation rate 30.2%) and 39 completed the questionnaire (response rate 97.5%). Salient consensus points included the following: the need to indicate workup studies from a sustained platelet count < 100 x 10^9^/L in the absence of a clear etiology; bone marrow aspiration in elderly patients with suspected ITP; beginning treatment in asymptomatic patients with a platelet count < 20 x 10^9^/L; not exceeding 6-7 weeks of corticosteroid therapy; switching from corticosteroids to one thrombopoietin receptor agonist (TRA); switching to other TRA or other options as combinations of them with immunosuppressive drugs in case of failure; how to reduce tapering TRA; treating patients with symptomatic persistent ITP and platelet count > 20 x 10^9^/L; and considering mucosal or severe bleeding as a basic criterion for hospital admission.

**Conclusions:**

The present consensus document provides a reference framework for the management of patients with ITP in clinical practice.

## 1. Introduction

Immune thrombocytopenia (ITP) is an acquired autoimmune disorder defined by isolated thrombocytopenia in the absence of other conditions associated with thrombocytopenia. Given the variability of ITP in clinical presentation, symptoms, and clinical course, the diagnosis relies on the exclusion of an alternative etiology for thrombocytopenia [1–4]. Regarding the pathogenesis, thrombocytopenia seems to be the result of dysregulation of the immune response, including the presence of antiplatelet antibodies, platelet destruction mediated by T-cells and the reticuloendothelial system, and impaired megakaryocyte function. The involvement of these pathogenic mechanisms may vary in the individual patient [2, 5–7].

Recommendations on standardization of terminology, definitions, and criteria of response of ITP were published in 2009 [8]. Different consensus documents for the diagnosis and treatment of ITP published subsequently [9–12] have been of remarkable value to reduce heterogeneity in the classification of patients and to improve the design and interpretation of results of clinical trials. Also, the reduced relevance of the platelet count in the occurrence of specific bleeding symptoms has contributed to improve the management of the disease by reducing adverse effects related to inadequate treatments [13, 14]. The development of drugs that can delay or even avoid the need of performing a splenectomy, such as thrombopoietin receptor agonists (TRAs), romiplostim, and eltrombopag [15–18], has been crucial to decrease morbidity, historically related to infections and bleeding.

ITP is a heterogeneous disease whose evolution and response to treatment is unpredictable at diagnosis [19]. The availability of new therapeutic options has raised new questions in the current approach to ITP management, introducing considerable variability in clinical practice. A recent multicenter study carried out in 15 Spanish hospitals showed remarkable differences in diagnosis and treatment of patients with ITP [20]. Thus, unanswered questions remain, such as how to identify patients who need treatment since many are asymptomatic despite thrombocytopenia or, in case of need for treatment, how to select the most appropriate option. In most cases an individualized approach is required considering platelet count, presence of bleeding, lifestyle, and other patient characteristics as well as potential adverse effects of treatment.

In order to clarify uncertainties about patient management and to select the most adequate treatment, a study using the Delphi method has been carried out on different aspects associated with the diagnosis of ITP, first- and second-line treatments, followup, and therapeutic approach in special settings. The objective of this project was to assess the level of agreement among expert hematologists regarding the best clinical practices for the diagnosis and management of patients with ITP.

## 2. Materials and Methods

In order to establish the present recommendations on the management of ITP patients in daily clinical practice, a consultation was made to a group of hematologists experts in the care of patients with ITP, members of the Spanish ITP Group (GEPTI) of the Spanish Society of Hematology and Hemotherapy (SEHH) routinely manage. Different aspects of the disease that in the hematologists' opinion should be considered in the study and treatment of patients with ITP were considered. A modified Delphi method was used to reach consensus. The original Delphi method involves three or more rounds, whereas the modified technique is limited to two rounds to avoid losses of acceptable response rates due to prolonged duration of the process and the negative influence on the interest of the panelists.

At the beginning of the project, a Scientific Committee composed of 11 specialists with proven experience and interest in the study of ITP was established. The scientific committee was responsible for the development of a 90-item questionnaire on ITP. Questions were grouped into eight sections: diagnosis (4 items), first-line treatment (11 items), second-line treatment (12 items), persistent ITP and refractory patients (16 items), followup (12 items), pregnant patients (12 items), safety and management of emergency and surgery (14 items), and secondary ITP (3 items). Each question was formulated so that it could be answered using a 4-point Likert scale, 1 = “never”, 2 = “sometimes”, 3 = “frequently”, and 4 = “always” according to the participant's opinion regarding what should be performed in clinical practice.

The questionnaire was sent to a total of 129 hematologists who were members of the GEPTI. At the end of the first round, participants' responses were collected and sent to the members of the Scientific Committee for analysis. Subsequently, a meeting was organized with the members of the Scientific Committee to share and discuss the results obtained. In this meeting and based on the consensus reached in the first round, it was decided that the answers obtained were sufficiently adequate to be included in the final study questionnaire.

Only fully completed questionnaires were considered for analysis. For each item, the median value of the four possible responses in the 4-point Likert scale was calculated. Considering median values, questions with a higher proportion of high frequency responses (“frequently” and “always”) were grouped as opposed to those of low frequency (“sometimes” and “never”) to facilitate the analysis. Consensus was established in favor of the recommendation when the sum of the responses “frequently” (Likert score 3) and “always” (Likert score 4) was equal to or greater than two thirds (66.6%) of the total responses obtained for that item. By contrast, consensus against the recommendation was reached when the sum of responses “sometimes” (Likert score 2) and “never” (Likert score 1) was equal to or greater than 66.6% of the total responses obtained for that item. When none of these previous assumptions were met, consensus neither in favor nor against the statement was reached.

## 3. Results

### 3.1. Characteristics of Participants

Of the 129 experts to whom the questionnaire was sent, 40 participated in the study (participation rate 30.2%) and 39 fully completed the questionnaire (response rate 97.5%). A total of 57.5% of participants reported a professional experience between 5 and 20 years in the management of ITP (10% less than 5 years and 17.5% more than 20 years), and 60% had an annual number of newly diagnosed patients between 5 and 15. The number of specialists in their respective hospital services varied between three and four in 30.7% of the cases, being greater than four in 28.2%. Also, 94% of the respondents reported freedom to prescribe any medication necessary for the management of patients with ITP.

### 3.2. Diagnosis

**Figure 1:**
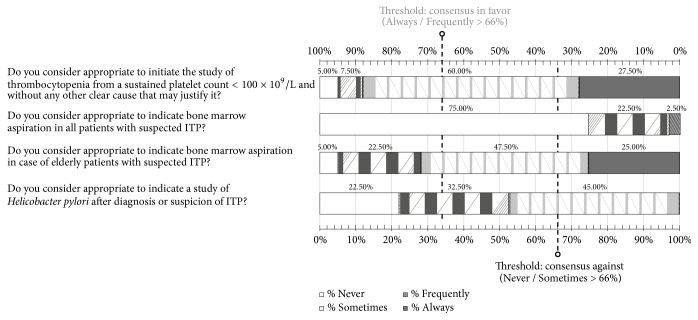
Results obtained in the section of the questionnaire regarding diagnosis of ITP. Distribution of the percentages of responses.

In this section of the questionnaire, consensus was reached in three of the four items (Figure 1). Experts agreed on the need to indicate workup studies in the presence of a sustained platelet count < 100 x 10^9^/L in the absence of any clear causative condition for the decreased platelet count. Also, consensus was reached in favor of performing bone marrow aspiration in elderly patients with suspicion of ITP. We considered people older than 65 years old elderly or fragile. By contrast, participants did not consider performing bone marrow aspiration in all patients with suspicion of ITP adequate. In addition, regarding the need for screening* Helicobacter pylori *infection in case of suspicion or diagnosis of ITP, 45% of respondents considered that screening should be performed and 55% considered that screening should only be performed in some cases or never. Details of the results obtained in each section of the questionnaire are shown in [Supplementary-material supplementary-material-1] of the Supplementary Material.

### 3.3. First-Line Treatment

**Figure 2:**
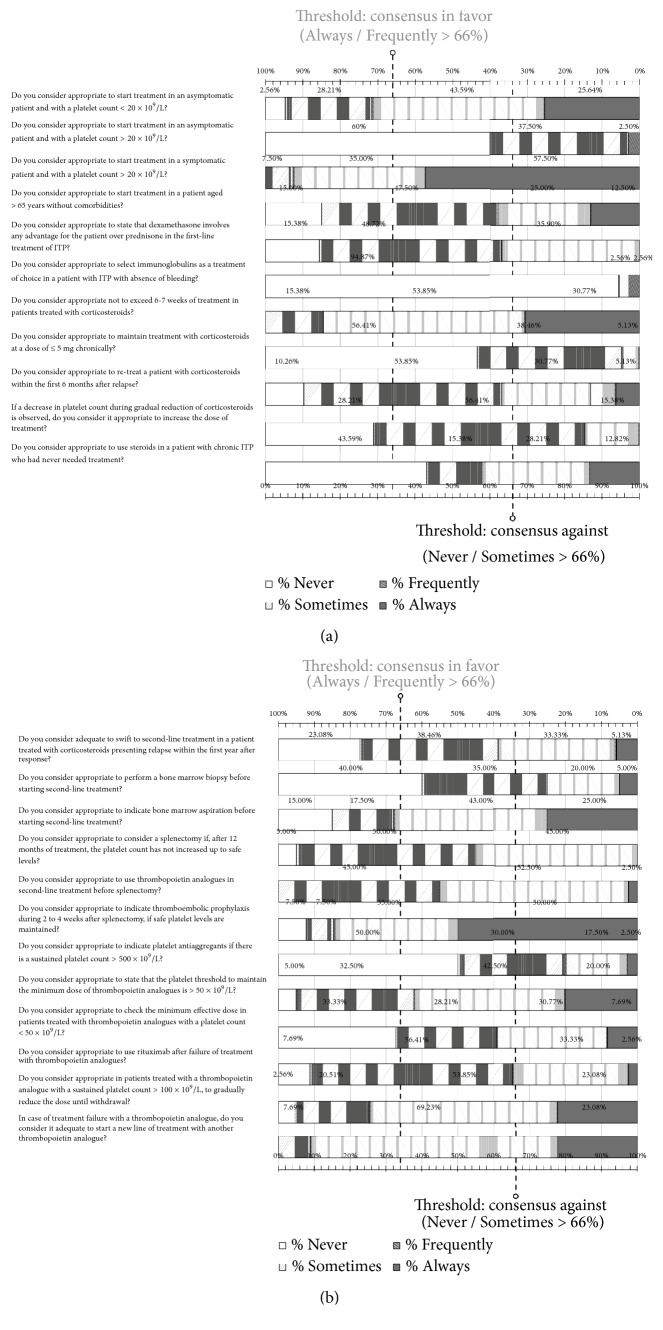
Results obtained in the section of the questionnaire regarding first-line treatment (a) and second-line treatment (b) of ITP. Distribution of the percentages of responses.

In this section that included 11 items (Figure 2(a)), consensus was reached for 7 questions. Participants coincided in starting treatment in asymptomatic patients with a platelet count < 20 x 10^9^/l, being indispensable in patients with symptoms although at higher platelet counts. Also, they agreed in favor of not exceeding 6-7 weeks of treatment in patients treated with corticosteroids. There was consensus against starting treatment in asymptomatic patients with a platelet count > 20 x 10^9^/L, maintaining the chronic use of corticosteroids at doses of ≤ 5 mg, prescribing immunoglobulins as the treatment of choice in the absence of bleeding, and increasing the dose of treatment in the presence of a decrease in platelet count during discontinuation of corticosteroids. Finally, no consensus was reached in starting treatment in a patient > 65 years of age without comorbidities, regarding the statement that dexamethasone implies some advantage over prednisone, resuming treatment with corticosteroids within the first 6 months after relapse, and in the use of corticosteroids in a patient with chronic ITP that had never needed treatment before.

### 3.4. Second-Line Treatment

In this section that included 12 items (Figure 2(b)), consensus was reached in 7 questions and there was lack of consensus in 5. 97,5% of the participants considered TPO-RA as the treatment of choice in second-line in comparison with other therapeutic options. Participants agreed to recommend bone marrow aspiration before starting second-line treatment and prescribe thromboembolic prophylaxis after splenectomy during a 2-4 weeks' period while maintaining safety platelet level. In case of treatment failure with a TRA, they considered switching to another TRA adequate. On the other hand, they agreed against performing a bone marrow biopsy before the switch, as well as prescribing antiplatelet agents' therapy in patients with a sustained platelet count > 500 x 10^9^/L. They also stated that, in patients treated with TRAs who had maintained a platelet count > 100 x 10^9^/L, it would be advisable to progressively reduce the treatment doses until withdrawal. Regarding the questions in which there was no consensus ([Supplementary-material supplementary-material-1] Supplementary Material), 56% of participants considered using rituximab in third line after treatment failure of TRA appropriate.

### 3.5. Persistent ITP and Refractory Patients

**Figure 3:**
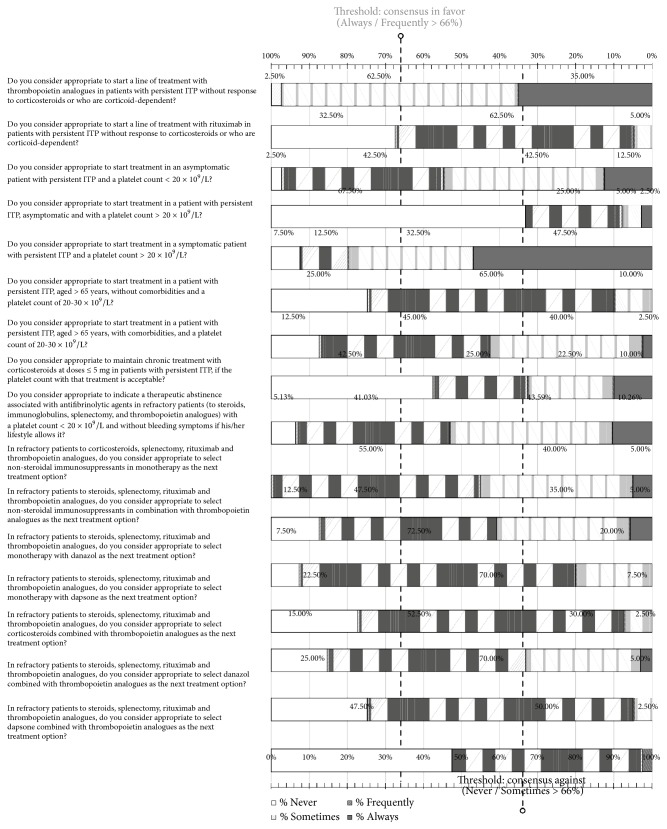
Results obtained in the section of the questionnaire regarding persistent ITP and refractory patients. Distribution of the percentages of responses.

In this section that included 16 items, consensus was obtained in 11 questions, with a lack of consensus in the remaining 5. In relation to patients with persistent ITP, it was considered adequate that patients with symptoms and platelet count > 20 x 10^9^/L should be treated, and it is necessary to begin treatment with TRA if they were either unresponsive to corticosteroids or corticosteroid-dependent, whereas it was not considered adequate to use rituximab. Also, it was not considered adequate to maintain prolonged corticosteroids treatment at doses of ≤ 5 mg, even in the presence of an acceptable platelet count. In those items in which consensus against the statement was obtained, the recommendation of not using monotherapy with danazol or dapsone, or in combination with TPO-RA, in refractory patients to corticosteroids, splenectomy, rituximab, and TRA agents should be noted. Moreover, consensus against starting treatment in a patient with persistent ITP, asymptomatic, and with a platelet count > 20 x 10^9^/l was reached, as well as in a patient with persistent ITP and older than 65 years of age, without comorbidities, and with a platelet count of 20-30 x 10^9^/L. On the other hand, 54% of participants reported that abstaining from treatment associated with antifibrinolytic agents may be considered in refractory patients with a platelet count < 20 x 10^9^/L without bleeding symptoms, if the patient's lifestyle allows for this decision (Figure 3). Details of the percentages of response for each question are shown in [Supplementary-material supplementary-material-1] of the Supplementary Material.

### 3.6. Followup

**Figure 4:**
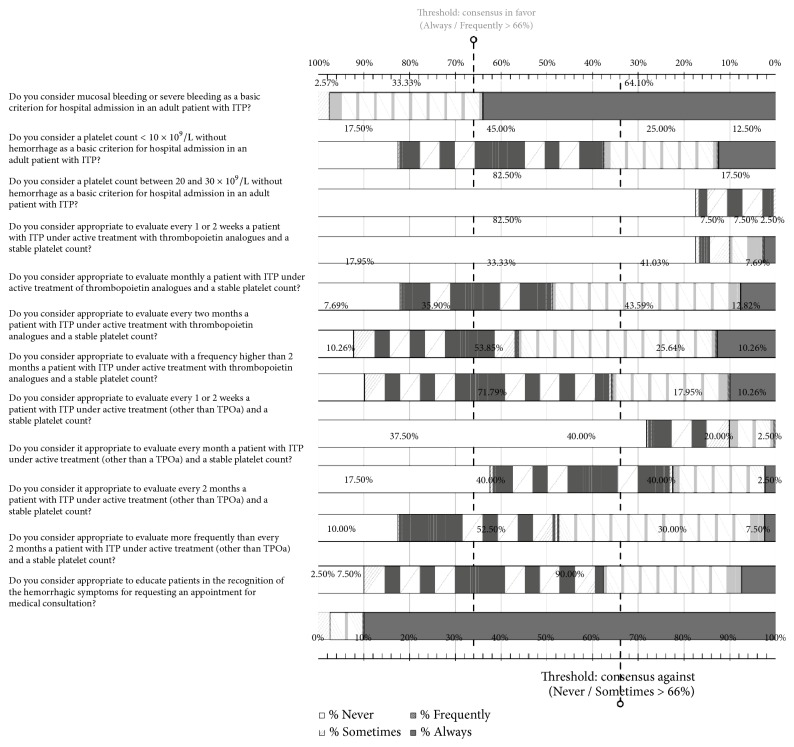
Results obtained in the section of the questionnaire regarding the follow-up of patients with ITP. Distribution of the percentages of responses.

In this section of 12 items, specialists reached consensus in 6 questions and there was no consensus in the remaining 6 (Figure 4). Mucosal or severe bleeding was considered a basic criterion for admission to the hospital. They also agreed on the need to educate patients in the recognition of bleeding manifestations. They did not consider that a platelet count < 10 x 10^9^/L or between 20 and 30 x 10^9^/L, in the absence of bleeding, was a criterion for hospitalization. Participants discarded to make specific recommendations regarding the frequency of visits based on the disease and preferred to schedule consultations according to the individual characteristics of each patient ([Supplementary-material supplementary-material-1], Supplementary Material).

### 3.7. Pregnancy

**Figure 5:**
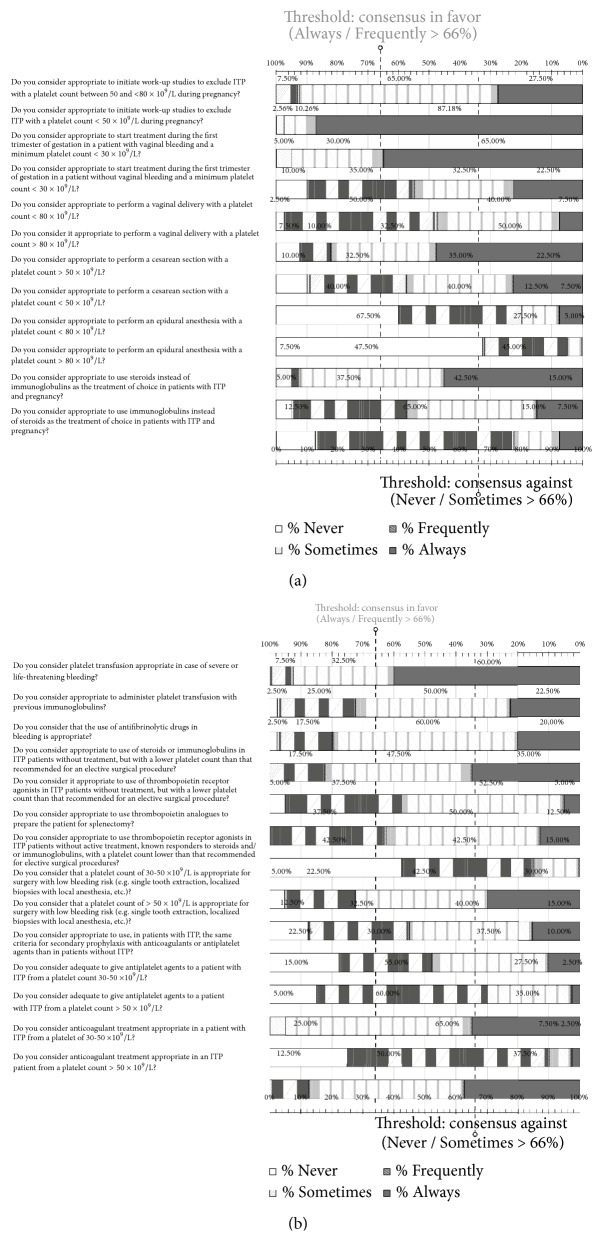
Results obtained in the section of the questionnaire regarding the management of patients with ITP during pregnancy (a) and in aspects related to emergencies, surgery, and safety (b). Distribution of the percentages of responses.

In the 12 items related to the management of patients with ITP during pregnancy, consensus was reached in 7 items. In the remaining 5 items, consensus was not obtained. Participants agreed to establish a minimum platelet count of 80 x 10^9^/L to perform a vaginal delivery and epidural anesthesia and did not consider it adequate to carry out a cesarean section with a platelet count < 50 x 10^9^/L or epidural anesthesia in patients with a platelet count < 80 x 10^9^/L. In pregnant patients with platelet counts between 50 and 80 x 10^9^/L, workup studies to exclude the diagnosis of ITP were recommended, being indispensable in patients with a platelet count < 50 x 10^9^/L. Also, there was an agreement regarding the importance of starting treatment in patients without hemorrhagic diathesis and a minimum platelet count of 30 x 10^9^/L even during the first trimester of pregnancy (Figure 5(a)) ([Supplementary-material supplementary-material-1], Supplementary Material).

### 3.8. Emergencies, Surgery, and Safety

In this section of the questionnaire that included 14 items, consensus was reached in 10 items, whereas in the remaining 4, consensus was not obtained (Figure 5(b)). Participants believed that platelet transfusion was essential in cases of severe bleeding or life-threatening hemorrhage, as well as the administration of platelets with previous immunoglobulins and the use of antifibrinolytic agents in hemorrhage. Participant also agreed to establish a minimum platelet count of 50 x 10^9^/L to indicate treatment with antiplatelet agents and anticoagulants and between 30 and 50 x 10^9^/L for surgical procedures of low hemorrhagic risk. In untreated patients with ITP and platelet count lower than that required for elective surgery, the use of corticosteroids or immunoglobulins was recommended. In addition, the use of TRA drugs was recommended in untreated patients, who have been responsive to corticosteroids or immunoglobulins, with a platelet count lower than that established for elective surgery ([Supplementary-material supplementary-material-1], Supplementary Material).

### 3.9. Secondary Immune Thrombocytopenia

**Figure 6:**
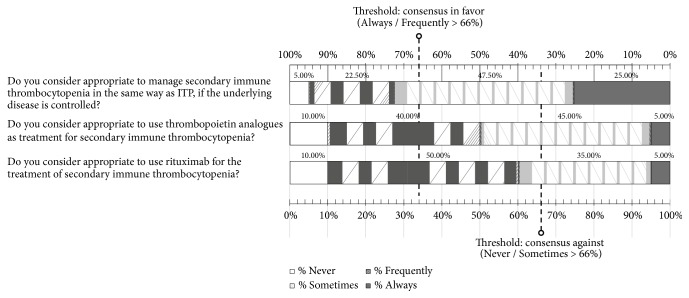
Results obtained in the section of the questionnaire regarding secondary immune thrombocytopenia. Distribution of the percentages of responses.

In this section of 3 items, participants recommended to manage secondary immune thrombocytopenia in the same way as ITP provided that the underlying disease was controlled (Figure 6) ([Supplementary-material supplementary-material-1], Supplementary Material).

## 4. Discussion

The aim of the Delphi method is to obtain an opinion, level of agreement, or consensus on a current topic or concern from among a group of specialists or experts. This is an iterative and anonymous process with controlled feedback and analysis of the results widely used in health sciences [21–23] Although the Delphi methodology has been applied in various hematology studies [24–28], no previous studies comprising a consensus of opinion have been published on ITP using the Delphi technique. With respect to the percentages of respondents having completed the rounds of questions set, the data varies depending on the characteristics of the study, including the number of experts, survey distribution methods, number of rounds, face-to-face meetings, etc. The participation rate in our study was 30.2%, but the percentage of participants having completed the Delphi rounds was 97.5%, being in the upper band of the range between 60% and 100% described in other similar studies published in the literature [22–28].

Different scientific societies and expert groups have developed guidelines and consensus documents with recommendations for the diagnosis and treatment of adult patients with ITP [10, 11, 29, 30]. However, agreement between recommendations and their application in real clinical practice continues to be insufficient [20, 31]. In the study by Lozano et al. [20] on the management of 101 adult patients with ITP, data collected from the medical records were compared with guidelines recommendations, and important inconsistencies in diagnosis and treatment were found. Regarding diagnosis, a cytological examination of the peripheral blood was never performed in 22.8% of the patients despite being recommended by all guidelines, whereas in more than one half of the patients (50.5%), a study of the bone marrow was carried out in the initial diagnosis even though this examination is not recommended routinely by the guidelines. In the present study, areas in which consensus was reached were generally in agreement with clinical practice guidelines, but there were some aspects in the clinical management of ITP that were not present in the guidelines and for which unified criteria are needed. Thus, participants agreed on the need that treatment with corticosteroids should not exceed 6-7 weeks, whereas in routine clinical practice, as published by Lozano et al. [20], the mean duration of treatment with corticosteroids was more than 70 days. In relation to the use of corticosteroids, it is interesting to note that whereas the study of Lozano et al. [20] revealed that 37.5% of patients received corticosteroids as second-line treatment, in the present consensus it was not considered appropriate to maintain chronic corticosteroid treatment at doses of ≤ 5 mg in patients with persistent ITP, even in the presence of an acceptable platelet count. In our study, participants denied the need of performing systematically bone marrow aspiration in patients with suspicion of ITP except in elderly patients. There is no study to recommend this practice in elderly patients but the risk of myelodiplastic syndrome in this group of patients is present.

Other interesting aspects in which consensus was reached refer to asymptomatic patients in whom starting treatment was established at a threshold platelet count of < 20 x 10^9^/L. In Spain, our real practice considers treatment in patients with less than 20,000 platelets, and we reflect that way of treatment in our national consensus of ITP treatment although we do not have our own publication to justify it. There are guidelines that indicate the possibility of treating the patient in the absence of relevant bleeding if the platelet count is between 20 and 30 x 10^9^/L [10, 32], while others do not recommend it [30, 33, 34]. Bleeding events was the major criterion for the initiation of treatment according to the expert panel. Age and comorbidity are also criteria that may be considered in decision making, although the toxicities of classical treatments in older patients are higher and have a greater risk for bleeding. In patients with ITP who do not respond to corticosteroids or are steroid-dependent, most participants would administer a TRA agent.

There are other clinically relevant aspects in the current clinical practice scenario for which consensus was obtained and that are not included in the guidelines, such as the convenience of switching to a different TRA drug in the event of previous TRA failure and the possibility of using these agents in the context of the patient's preparation for surgery, in secondary ITP, or in combination with immunosuppressant drugs in multirefractory patients. Regarding combination therapy, there are case reports and a retrospective series of Mahévas et al. [35] with a favorable response in 70% of patients. On the other hand, the recommendation suggested in different studies seems also very interesting [33, 34], although predictors of sustained response in this setting remain unclear. Recently, some clinical practice experience has been published showing that patients treated with such agonists who maintain a platelet count higher than 100 x 10^9^/L for 3 months have a greater likelihood of success after discontinuation of treatment [36, 37].

Treatment of refractory ITP is a clinical challenge and frequently the results obtained are poor. In this study, no consensus was reached regarding the management of refractory patients to TRA, splenectomy, and rituximab [38].

Results of the present study should be interpreted considering that responses to the questionnaire reflect the specialists' views of what they would do in different scenarios posed by each question, and not necessarily what they do in real clinical practice. However, the Delphi method allowed exploring systematically the management of adult patients with ITP based on the qualified opinion of physicians routinely treating these patients. The management of patients with ITP is rapidly evolving, and over the last 15 years, several novel treatments have improved practice, with many steroid-sparing agents and a significant reduction in the splenectomy rate. Although this has improved clinical care, many therapeutic challenges remain. There is no diagnostic test or biomarkers to direct treatment and there are few comparative studies to help management decisions.

## 5. Conclusions

This Delphi survey study conducted in a sample of hematologists members of the Spanish Immune Thrombocytopenia Group (GEPTI) of the Spanish Society of Hematology and Hemotherapy may offer a clear framework of the real management of adult patients with ITP in clinical practice.

## Data Availability

The data used to support the findings of this study are available from the corresponding author upon request.

## Supplementary Materials

Supplementary Materials Supplementary Table 1. Results of the different sections of the questionnaire.
