# Supplementary material for: Management of Adult Patients with Primary Immune Thrombocytopenia (ITP) in Clinical Practice: A Consensus Approach of the Spanish ITP Expert Group
Source: Adv Hematol. 2019 Aug 22;2019:4621416. doi: 10.1155/2019/4621416 (PMC6724426; doi:10.1155/2019/4621416)
Supplement: Supplementary Materials — Supplementary Table 1. Results of the different sections of the questionnaire. [file 4621416.f1.pdf]

Annex. Supplementary material

Supplementary Table 1. Results of the different sections of the questionnaire

Questionnaire section	Never %	Sometimes %	Frequently %	Always %	Mean	Median	Mode
**Diagnosis**							
– Do you consider appropriate to initiate the study of thrombocytopenia from a sustained platelet count < 100 x 10^9/L and without any other clear cause that may justify it?	5.0	7.5	60.0	27.5	3.100	Frequently	Frequently
– Do you consider appropriate to indicate bone marrow aspiration in case of elderly patients with suspected ITP?	5.0	22.5	47.5	25.0	2.925	Frequently	Frequently
– Do you consider appropriate to indicate bone marrow aspiration in all patients with suspected ITP?	75.0	22.5	2.5	0	1.275	Never	Never
– Do you consider appropriate to indicate a study of *Helicobacter pylori* after diagnosis or suspicion of ITP?	22.5	32.5	45.0	0	2.225	Sometimes	Frequently
**First-line treatment**							
– Do you consider appropriate to start treatment in an asymptomatic patient and with a platelet count < 20 x 10^9/L?	2.6	28.2	43.6	25.6	2.923	Frequently	Frequently
– Do you consider appropriate to start treatment in a symptomatic patient and with a platelet count > 20 x 10^9/L?	0	7.5	35.0	57.5	3.500	Always	Always
– Do you consider appropriate not to exceed 6-7 weeks of treatment in patients treated with corticosteroids?	0	15.4	53.8	30.8	3.154	Frequently	Frequently
– Do you consider appropriate to start treatment in an asymptomatic patient and with a platelet count > 20 x10^9/L?	60.0	37.5	2.5	0	1.425	Never	Never

Questionnaire section	Never %	Sometimes %	Frequently %	Always %	Mean	Median	Mode
**First-treatment (continued)**							
– Do you consider appropriate to start treatment in a patient aged > 65 years without comorbidities?	15.0	47.5	25.0	12.5	2.350	Sometimes	Sometimes
– Do you consider appropriate to state that dexamethasone involves any advantage for the patient over prednisone in the first-line treatment of ITP?	15.4	48.7	35.9	0	2.205	Sometimes	Sometimes
– Do you consider appropriate to maintain treatment with corticosteroids at a dose of ≤ 5 mg chronically?	56.4	38.5	5.1	0	1.487	Never	Never
– Do you consider appropriate to select immunoglobulins as a treatment of choice in a patient with ITP with absence of bleeding?	94.9	2.6	2.6	0	1.077	Never	Never
– Do you consider appropriate to re-treat a patient with corticosteroids within the first 6 months after relapse?	10.3	53.8	30.8	5.1	2.308	Sometimes	Sometimes
– If a decrease in platelet count during gradual reduction of corticosteroids is observed, do you consider it appropriate to increase the dose of treatment?	28.2	56.4	15.4	0	1.872	Sometimes	Sometimes
– Do you consider appropriate to use steroids in a patient with chronic ITP who had never needed treatment?	43.6	15.4	28.2	12.8	2.103	Sometimes	Never
**Second-line treatment**							
– Do you consider appropriate to indicate bone marrow aspiration before starting second-line treatment?	15.0	17.5	42.5	25.0	2.775	Frequently	Frequently
– Do you consider appropriate to use thrombopoietin analogues in second-line treatment before splenectomy?	0	45.0	52.5	2.50	2.575	Frequently	Frequently
– Do you consider appropriate to indicate thromboembolic prophylaxis during 2 to 4 weeks after splenectomy, if safe platelet levels are maintained?	7.5	7.5	35.0	50.0	3.275	Frequently - Always	Always
**Second-line treatment (continued)**							

Questionnaire section	Never %	Sometimes %	Frequently %	Always %	Mean	Median	Mode
– Do you consider appropriate to state that the platelet threshold to maintain the minimum dose of thrombopoietin analogues is > 50 x 10^9/L?	5.0	32.5	42.5	20.0	2.775	Frequently	Frequently
– Do you consider appropriate in patients treated with a thrombopoietin analogue with a sustained platelet count > 100 x 10^9/L, to gradually reduce the dose until withdrawal?	2.6	20.5	53.8	23.1	2.974	Frequently	Frequently
– In case of treatment failure with a thrombopoietin analogue, do you consider it adequate to start a new line of treatment with another thrombopoietin analogue?	0	7.7	69.2	23.1	3.154	Frequently	Frequently
– Do you consider adequate to swift to second-line treatment in a patient treated with corticosteroids presenting relapse within the first year after response?	23.1	38.5	33.3	5.1	2.205	Sometimes	Sometimes
– Do you consider appropriate to perform a bone marrow biopsy before starting second-line treatment?	40.0	35.0	20.0	5.0	1.900	Sometimes	Never
– Do you consider appropriate to consider a splenectomy if, after 12 months of treatment, the platelet count has not increased up to safe levels?	5.0	50.0	45.0	0	2.400	Sometimes	Sometimes
– Do you consider appropriate to indicate platelet antiaggregants if there is a sustained platelet count > 500 x 10^9/L?	50.0	30.0	17.5	2.5	1.725	Never - Sometimes	Never
– Do you consider appropriate to check the minimum effective dose in patients treated with thrombopoietin analogues with a platelet count < 50 x 10^9/L?	33.3	28.2	30.8	7.7	2.128	Sometimes	Never
– Do you consider appropriate to use rituximab after failure of treatment with thrombopoietin analogues?	7.7	56.4	33.3	2.7	2.308	Sometimes	Sometimes
**PERSISTENT ITP AND REFRACTORY PATIENTS**							
– Do you consider appropriate to start a line of treatment with thrombopoietin analogues in patients with	0	2.5	62.5	35.0	3.325	Frequently	Frequently

Questionnaire section	Never %	Sometimes %	Frequently %	Always %	Mean	Median	Mode
persistent ITP without response to corticosteroids or who are corticoid-dependent?							
– Do you consider appropriate to start treatment in an asymptomatic patient with persistent ITP and a platelet count < 20 $x10^9/L$?	2.5	42.5	42.5	12.5	2.650	Frequently	Sometimes
– Do you consider appropriate to start treatment in a symptomatic patient with persistent ITP and a platelet count > 20 $x10^9/L$?	7.5	12.5	32.5	47.5	3.20	Frequently	Always
– Do you consider appropriate to indicate a therapeutic abstinence associated with antifibrinolytic agents in refractory patients (to steroids, immunoglobulins, splenectomy, and thrombopoietin analogues) with a platelet count < 20 x $10^9/L$ and without bleeding symptoms if his/her lifestyle allows it?	5.1	41.0	43.6	10.3	2.590	Frequently	Frequently
– Do you consider appropriate to start a line of treatment with rituximab in patients with persistent ITP without response to corticosteroids or who are corticoid-dependent?	32.5	62.5	5.0	0	1.725	Sometimes	Sometimes
– Do you consider appropriate to start treatment in a patient with persistent ITP, asymptomatic and with a platelet count > 20 $x10^9/L$?	67.5	25.0	5.0	2.5	1.425	Never	Never
– Do you consider appropriate to start treatment in a patient with persistent ITP, aged > 65 years, without comorbidities and a platelet count of 20-30 x $10^9/L$?	25.0	65.0	10.0	0	1.850	Sometimes	Sometimes
– Do you consider appropriate to start treatment in a patient with persistent ITP, aged > 65 years, with comorbidities, and a platelet count of 20-30 x $10^9/L$?	12.5	45.0	40.0	2.5	2.325	Sometimes	Sometimes
– Do you consider appropriate to maintain chronic treatment with corticosteroids at doses ≤ 5 mg in patients with persistent ITP, if the platelet count with that treatment is acceptable?	42.5	25.0	22.5	10.0	2.000	Sometimes	Never

Questionnaire section	Never %	Sometimes %	Frequently %	Always %	Mean	Median	Mode
– In refractory patients to corticosteroids, splenectomy, rituximab and thrombopoietin analogues, do you consider appropriate to select non-steroidal immunosuppressants in monotherapy as the next treatment option?	0	55.0	40.0	5.0	2.500	Sometimes	Sometimes
– In refractory patients to steroids, splenectomy, rituximab and thrombopoietin analogues, do you consider appropriate to select non-steroidal immunosuppressants in combination with thrombopoietin analogues as the next treatment option?	12.5	47.5	35.0	5.0	2.325	Sometimes	Sometimes
– In refractory patients to steroids, splenectomy, rituximab and thrombopoietin analogues, do you consider appropriate to select monotherapy with danazol as the next treatment option?	7.5	72.5	20.0	0	2.125	Sometimes	Sometimes
– In refractory patients to steroids, splenectomy, rituximab and thrombopoietin analogues, do you consider appropriate to select monotherapy with dapsone as the next treatment option?	22.5	70.0	7.5	0	1.850	Sometimes	Sometimes
– In refractory patients to steroids, splenectomy, rituximab and thrombopoietin analogues, do you consider appropriate to select corticosteroids combined with thrombopoietin analogues as the next treatment option?	15.0	52.5	30.0	2.5	2.200	Sometimes	Sometimes
– In refractory patients to steroids, splenectomy, rituximab and thrombopoietin analogues, do you consider appropriate to select danazol combined with thrombopoietin analogues as the next treatment option?	25.0	70.0	5.0	0	1.800	Sometimes	Sometimes
– In refractory patients to steroids, splenectomy, rituximab and thrombopoietin analogues, do you consider appropriate to select dapsone combined with	47.5	50.0	2.5	0	1.550	Sometimes	Sometimes

Questionnaire section	Never %	Sometimes %	Frequently %	Always %	Mean	Median	Mode
thrombopoietin analogues as the next treatment option?							
**Follow-up**							
– Do you consider mucosal bleeding or severe bleeding as a basic criterion for hospital admission in an adult patient with ITP?	0	2.6	33.3	64.1	3.615	Always	Always
– Do you consider appropriate to educate patients in the recognition of the hemorrhagic symptoms for requesting an appointment for medical consultation?	0	2.5	7.5	90.0	3.875	Always	Always
– Do you consider appropriate to evaluate every two months a patient with ITP under active treatment with thrombopoietin analogues and a stable platelet count?	7.7	35.9	43.6	12.8	2.615	Frequently	Frequently
– Do you consider a platelet count <10 x 10^9/L without hemorrhage as a basic criterion for hospital admission in an adult patient with ITP?	17.5	45.0	25.0	12.5	2.325	Sometimes	Sometimes
– Do you consider a platelet count between 20 and 30 x 10^9/L without hemorrhage as a basic criterion for hospital admission in an adult patient with ITP?	82.5	17.5	0	0	1.175	Never	Never
– Do you consider appropriate to evaluate every 1 or 2 weeks a patient with ITP under active treatment with thrombopoietin analogues and a stable platelet count?	82.5	7.5	7.5	2.5	1.300	Never	Never
– Do you consider appropriate to evaluate monthly a patient with ITP under active treatment of thrombopoietin analogues and a stable platelet count?	17.9	33.3	41.0	7.7	2.385	Sometimes	Frequently
– Do you consider appropriate to evaluate with a frequency higher than 2 months a patient with ITP under active treatment with thrombopoietin analogues and a stable platelet count?	10.3	53.8	25.6	10.7	2.359	Sometimes	Sometimes

Questionnaire section	Never %	Sometimes %	Frequently %	Always %	Mean	Median	Mode
Follow-up (continued)							
– Do you consider appropriate to evaluate every 1 or 2 weeks a patient with ITP under active treatment (other than TPOa) and a stable platelet count?	71.8	17.9	10.3	0	1.385	Never	Never
– Do you consider it appropriate to evaluate every month a patient with ITP under active treatment (other than a TPOa) and a stable platelet count?	37.5	40.0	20.0	2.5	1.875	Sometimes	Sometimes
– Do you consider it appropriate to evaluate every 2 months a patient with ITP under active treatment (other than TPOa) and a stable platelet count?	17.5	40.0	40.0	2.5	2.275	Sometimes	Sometimes
– Do you consider appropriate to evaluate more frequently than every 2 months a patient with ITP under active treatment (other than TPOa) and a stable platelet count?	10.0	52.5	30.0	7.5	2.350	Sometimes	Sometimes
**Pregnancy**							
– Do you consider appropriate to initiate work-up studies to exclude ITP with a platelet count between 50 and <80 x 10^9/L during pregnancy?	0	7.5	65.0	27.5	3.200	Frequently	Frequently
– Do you consider appropriate to initiate work-up studies to exclude ITP with a platelet count < 50 x 10^9/L during pregnancy?	0	2.6	10.3	87.2	3.846	Always	Always
– Do you consider appropriate to start treatment during the first trimester of gestation in a patient with vaginal bleeding and a minimum platelet count < 30 x 10^9/L?	0	5.0	30.0	65.0	3.600	Always	Always
– Do you consider appropriate to start treatment during the first trimester of gestation in a patient without vaginal bleeding and a minimum platelet count < 30 x 10^9/L?	10.0	35.0	32.5	22.5	2.675	Frequently	Sometimes

Questionnaire section	Never %	Sometimes %	Frequently %	Always %	Mean	Median	Mode
Pregnancy (continued)							
– Do you consider it appropriate to perform a vaginal delivery with a platelet count > 80 x 10^9/L?	7.50	10.0	32.5	50.0	3.250	Frequently - Always	Always
– Do you consider appropriate to perform a cesarean section with a platelet count > 50 x 10^9/L?	10.0	32.50	35.0	22.5	2.700	Frequently	Frequently
– Do you consider appropriate to perform an epidural anesthesia with a platelet count > 80 x 10^9/L?	0	7.50	47.5	45.0	3.375	Frequently	Frequently
– Do you consider appropriate to use steroids instead of immunoglobulins as the treatment of choice in patients with ITP and pregnancy?	5.0	37.50	42.5	15.0	2.675	Frequently	Frequently
– Do you consider appropriate to perform a vaginal delivery with a platelet count < 80 x 10^9/L?	2.5	50.0	40.0	7.5	2.525	Sometimes	Sometimes
– Do you consider appropriate to perform a cesarean section with a platelet count < 50 x 10^9/L?	40.0	40.0	12.5	7.5	1.875	Sometimes	Never
– Do you consider appropriate to perform an epidural anesthesia with a platelet count < 80 x 10^9/L?	67.5	27.5	5.0	0	1.375	Never	Never
– Do you consider appropriate to use immunoglobulins instead of steroids as the treatment of choice in patients with ITP and pregnancy?	12.5	65.0	15.0	7.5	2.175	Sometimes	Sometimes
**Emergencies, surgery, and safety**							
– Do you consider platelet transfusion appropriate in case of severe or life-threatening bleeding?	0	7.5	32.5	60.0	3.525	Always	Always
– Do you consider appropriate to administer platelet transfusion with previous immunoglobulins?	2.5	25.0	50.0	22.5	2.925	Frequently	Frequently
– Do you consider that the use of antifibrinolytic drugs in bleeding is appropriate?	2.5	17.5	60.0	20.0	2.975	Frequently	Frequently
– Do you consider appropriate to use of steroids or immunoglobulins in ITP patients without treatment, but with a lower platelet count than that recommended for	0	17.5	47.5	35.0	3.175	Frequently	Frequently

Questionnaire section	Never %	Sometimes %	Frequently %	Always %	Mean	Median	Mode
an elective surgical procedure?							
– Do you consider it appropriate to use of thrombopoietin receptor agonists in ITP patients without treatment, but with a lower platelet count than that recommended for an elective surgical procedure?	5.0	37.5	52.5	5.0	2.575	Frequently	Frequently
– Do you consider appropriate to use thrombopoietin analogues to prepare the patient for splenectomy?	0	37.5	50.0	12.5	2.750	Frequently	Frequently
– Do you consider that a platelet count of 30-50 x 10^9/L is appropriate for surgery with low bleeding risk (e.g. single tooth extraction, localized biopsies with local anesthesia, etc.)?	5.0	22.5	42.5	30.0	2.975	Frequently	Frequently
– Do you consider that a platelet count of > 50 x 10^9/L is appropriate for surgery with low bleeding risk (e.g. single tooth extraction, localized biopsies with local anesthesia, etc.)?	12.5	32.5	40.0	15.0	2.575	Frequently	Frequently
– Do you consider adequate to give antiplatelet agents to a patient with ITP from a platelet count > 50 x 10^9/L?	0.00	5.0	60.0	35.0	3.300	Frequently	Frequently
– Do you consider anticoagulant treatment appropriate in an ITP patient from a platelet count > 50 x 10^9/L?	0	12.5	50.0	37.5	3.250	Frequently	Frequently
– Do you consider appropriate to use thrombopoietin receptor agonists in ITP patients without active treatment, known responders to steroids and/or immunoglobulins, with a platelet count lower than that recommended for elective surgical procedures?	42.5	42.5	15.0	0.0	1.725	Sometimes	Never
– Do you consider appropriate to use, in patients with ITP, the same criteria for secondary prophylaxis with anticoagulants or antiplatelet agents than in patients without ITP?	22.5	30.0	37.5	10.0	2.350	Sometimes	Frequently
– Do you consider adequate to give antiplatelet agents to a patient with ITP from a platelet count 30-50 x 10^9/L?	15.0	55.0	27.5	2.5	2.175	Sometimes	Sometimes

Questionnaire section	Never %	Sometimes %	Frequently %	Always %	Mean	Median	Mode
Emergencies, surgery, and safety (continued)							
– Do you consider anticoagulant treatment appropriate in a patient with ITP from a platelet of 30-50 x 10^9/L?	25.0	65.0	7.5	2.5	1.875	Sometimes	Sometimes
**Secondary immune thrombocytopenia**							
– Do you consider appropriate to manage secondary immune thrombocytopenia in the same way as ITP, if the underlying disease is controlled?	5.0	22.5	47.5	25.0	2.925	Frequently	Frequently
– Do you consider appropriate to use thrombopoietin analogues as treatment for secondary immune thrombocytopenia?	10.0	40.00	45.0	5.0	2.450	Sometimes - frequently	Frequently
– Do you consider appropriate to use rituximab for the treatment of secondary immune thrombocytopenia?	10.0	50.0	35.0	5.0	2.350	Sometimes	Sometimes

Never: 1; sometimes: 2: frequently: 3; always: 4. TPOa: thrombopoietin analogues.
